# Caveolin-2 is regulated by BRD4 and contributes to cell growth in pancreatic cancer

**DOI:** 10.1186/s12935-020-1135-0

**Published:** 2020-02-18

**Authors:** Feng Jiao, Ting Han, Cuncun Yuan, Yiyi Liang, Jiujie Cui, Meng Zhuo, Liwei Wang

**Affiliations:** 1 Department of Oncology, Renji Hospital, School of Medicine, Shanghai Jiaotong University, 160 Pujian Road, Shanghai, 200127 China; 2 Department of Pathology, Fudan University Eye Ear Nose and Throat Hospital, 83 Fenyang Road, Shanghai, 201114 China

**Keywords:** Pancreatic cancer, BRD4, Caveolin-2, BET inhibitors

## Abstract

**Background:**

The bromodomain and extra-terminal domain (BET) family of proteins, especially BRD4 play an important role in epigenetic regulation, and are essential for cell survival and also are promising anticancer targets. This study aims to analyze the effect of BRD4 on the cell growth and progression of pancreatic cancer and novel mechanisms involved.

**Methods:**

Expression of BRD4 in pancreatic cancer and paired adjacent noncancerous tissues from 76 patients was analyzed by western blotting, immunohistochemistry, and real time PCR. Its correlation with the clinicopathological characteristics and prognosis of pancreatic cancer patients was analyzed. The effects of BRD4 on the cell proliferation were detected by colony formation assay and sulforhodamine B assay. Migration and invasion were determined by Transwell assays, and the effect of BRD4 on subcutaneous tumor formation was verified in nude mice. Cell cycle analysis was detected by flow cytometry. The potential downstream targets of BRD4 and related molecular mechanisms were clarified by RNA sequencing, chromatin immunoprecipitation and dual luciferase reporter assay.

**Results:**

BRD4 was overexpressed in pancreatic cancer. Biological results showed that BRD4 functioned as tumor promoter, facilitated cell proliferation, migration and invasion in vitro and in vivo. Further, caveolin-2 was selected as the downstream gene of BRD4 by RNA sequencing. Caveolin-2 overexpression can partially reverse the decreased cell growth ability caused by BRD4 knockdown, but did not affect cell migration and invasion. Chromatin immunoprecipitation assay and dual luciferase reporter assay revealed BRD4 could bind to the promoter region of caveolin-2 and upregulate caveolin-2 expression. Clinical data further indicated a positive correlation between BRD4 and caveolin-2 expression. BRD4 (high)/caveolin-2 (high) correlated with shorter overall survival of patients with pancreatic cancer. Multivariate analysis revealed that both BRD4 and caveolin-2 were independent factors.

**Conclusions:**

Our findings reveal the oncogenic effects of BRD4 in pancreatic cancer and elucidate a possible mechanism by which BRD4 and caveolin-2 act to enhance cell growth. Targeting the BRD4-caveolin-2 interaction by development of BET inhibitors will be a therapeutic strategy for pancreatic cancer.

## Background

Pancreatic cancer (PC) is a highly lethal disease, for which mortality closely parallels incidence. It is expected to become the second leading cause of tumor-related mortality in the United States by 2030 [1]. Despite rapid advances in molecular and therapeutic approaches [2], the prognosis of PC is poor, with a 5-year survival rate less than 8% [3]. Therefore, it is still necessary to explore the mechanism and clarify new targets.

Epigenetic regulation has become an alternative target for pancreas development and disease [4, 5]. By interacting with acetylated lysine residues, the bromodomain and extra-terminal domain (BET) family of receptor proteins play important roles in epigenetic regulation [6]. BET inhibition is considered as an effective anti-tumor treatment [7]. Our previous study [8] has revealed that the BET inhibitor JQ1 effectively suppressed vasculogenic mimicry of PC via the ERK1/2-MMP-2/9 signaling pathway both in vitro and in vivo. Among members of the BET family, BRD4 is one of the most widely expressed and studied genes and participates in cell growth and inflammation [9]. Recent studies have demonstrated that BRD4 plays an important role in tumor development and progression [10]. BRD4 is widely recognized for its role in the regulation of hyperenhancer tissue and oncogene expression [11]. Suppression of BRD4 facilitates communication between hyperenhancer and the target promoter, leading to cell-specific repression of oncogenes and cell death [11]. In fact, the non-transcriptional role of BRD4 in controlling DNA damage checkpoint activation and repair, as well as telomere maintenance, has been proposed, providing a new perspective on the multiple functions of the protein and opening up new prospects for BET inhibitor’s application in cancer [11]. However, whether or not BRD4 in PC is a tumor promoter or suppressor remains controversial. In the present study, we identified an essential role of BRD4 in maintaining PC growth and progression, and performed underlying mechanisms analysis.

## Materials and methods

### Ethics statement

Written consents were obtained from all subjects. This study was approved by the Research Ethics Committee of Renji Hospital, School of Medicine, Shanghai Jiaotong University and the first affiliated hospital with Nanjing Medical University. Written informed consent was obtained from all subjects.

### Patients and tissue samples

Seventy-six PC tissue samples and adjacent normal paraffin-embedded tissue samples were collected from the Department of Pathology, the first affiliated hospital with Nanjing Medical University, between January 2012 and December 2016. All of the patients were diagnosed and confirmed by histology. Tissue microarray (TMA) constructed by Shanghai Outdo Biotech Company (China), contained well-documented clinicopathological information, including age, gender, clinical stage, invasion depth, lymph nodes metastasis, distant metastasis, tumor differentiation, tumor location, nervous invasion, vessel invasion, and follow-up data (ended in December, 2018). Detailed information can be found in Additional file 1: Table S1.

Additionally, liquid nitrogen frozen PC tissue samples and adjacent normal tissues were obtained by surgical resection from ten patients at the department of bile and pancreatic surgery, Renji Hospital, School of Medicine, Shanghai Jiaotong University (160 Pujian Road, Shanghai 200127, China).

### Immunohistochemical staining

TMA were deparaffinized in xylene and rehydrated in a graded alcohol. 3% H_2_O_2_ block, and antigen retrieval was performed after heating in citrate buffer. The sections were incubated with an antibody against BRD4 (Cat. no. ab128874, 1:100; Abcam, Cambridge, MA USA) and CAV-2 (Cat. no. ab2911, 1:100; Abcam, Cambridge, MA USA) at 4 °C overnight and were incubated with biotinylated secondary antibodies.

### Scoring of immunohistochemistry

A double-blind scoring was performed independently by two investigators. BRD4 and CAV-2 expression were scored according to staining intensity and the percentage of positive cells as previously described [12]. The percentage of positive cells was scored as follows: 0% (0), 1–10% (1), 11–50% (2), 51–80% (3), 81–100% (4). Staining intensity was scored as follows: no staining (0), week (1), moderate (2), and strong (3). Comprehensive score = staining percentage × intensity. BRD4 or CAV-2 expression was classified as follows: < 6 low expression, ≥ 6 high expression.

### Cell culture

Human normal pancreatic ductal cells HPDE and PC cell lines AsPC-1, BxPC-3, CFPAC-1, PANC-1, MIAPaCa-2, SW1990 and SU.86.86 were all obtained from Chinese Academy of Sciences Cell Bank (Shanghai, China). All cell lines were last authenticated in January 2018 by polymorphic short tandem repeat analysis after a series of experiments, and used for a maximum of fifteen passages. SW1990 was cultured in L-15 medium supplemented with 10% fetal bovine serum (FBS, all purchased from Gibco, Grand Island, NY, USA), and grown in room temperature air. PANC-1 was cultured in DMEM supplemented with 10% FBS, MIAPaCa-2 in DMEM supplemented with 10% FBS and 5% horse serum, the other cells in RPMI-1640 supplemented with 10% FBS, grown at 37 °C in 5% CO_2_ and saturated humidity.

### Plasmids transfection

BRD4 nc/siRNA/control/overexpression, as well as CAV-2 nc/siRNA/control/overexpression were all designed and purchased from GeneChem (Shanghai, China). SiRNA targeting BRD4 (BRD4-si1, sense 5′-CACGGUACCAAACACAACUTT-3′, antisense 5′-AGUUGUGUUUGGUACCGUGTT-3′ and BRD-si2, sense 5′-CUACACGACUACUGUGACATT-3′, antisense 5′-UGUCACAGUAGUCGUGUAGTT-3′) was used to knockdown the expression of BRD4. And siRNA targeting CAV-2 (CAV-2-si1, sense 5′-GUGCAGACAAUAUGGAAGATT-3′, antisense 5′-GGCUCAACUCGCAUCUCAATT-3′ and CAV-2-si2, sense 5′-CUACACGACUACUGUGACATT-3′, antisense 5′-UUGAGAUGCGAGUUGAGCCTT-3′) was used to downregulate the CAV-2 expression. Negative control siRNA (4 µg; non‑targeting) and BRD4/CAV-2 siRNA (4 µg) were transiently transfected using 8 µl Lipofectamine 2000 (Invitrogen; Thermo Fisher Scientific, Inc.) according to manufacturer’s instructions. PANC-1 was infected with lentiviral particles containing specific or negative control vectors (GeneChem (Shanghai, China) and the polyclonal cells with puromycin resistance (1 µg/ml) were selected for further experiments. The short hairpin RNA sequences for targeting the BRD4 gene were as follows. BRD4-sh1, sense 5′-CcggCACGGTACCAAACACAACTcTCAAGAGAAGTTGTGTTTGGTACCGTGTTTTTTg-3′, antisense 5′-aattcaaaaaaCACGGTACCAAACACAACTTCTCTTGAgAGTTGTGTTTGGTACCGTG-3′. BRD4-sh2, sense 5′-CcggCTACACGACTACTGTGACAcTCAAGAGATGTCACAGTAGTCGTGTAGTTTTTTg-3′, antisense 5′-aattcaaaaaaCTACACGACTACTGTGACATCTCTTGAgTGTCACAGTAGTCGTGTAG-3′.

### RNA sequencing and data analysis for gene expression

According to the above steps, BRD4-nc and BRD4-si1 were transfected into PANC-1 cells. After verifying the siRNA efficiency of transient transfection, each group with three repetitions was sent to Shanghai Bio Technology Corporation for purification and sequencing analysis (Illumina HiSeq 2500, Illumina, USA).

Sequencing raw reads were preprocessed by filtering out rRNA reads, sequencing adapters, short-fragment reads and other low-quality reads. We used Tophat v2.1.0 [13] to map the cleaned reads to human genome 19 references with two mismatches. After genome mapping, Cufflinks v2.1.1 [14] was run with a reference annotation to generate FPKM values for known gene models. Differentially expressed genes were identified using Cuffdiff [14]. The p-value significance threshold in multiple tests was set by the false discovery rate (FDR) [15]. The fold-changes were also estimated according to the FPKM in each sample. The differentially expressed genes were selected using the following filter criteria: FDR ≤ 0.05 and fold-change ≥ 2.

### Real-time reverse transcription polymerase chain reaction (RT-qPCR)

Total RNA from cells or tissues was extracted using TRIzol^®^ reagent (Invitrogen; Thermo Fisher Scientific, Inc.). The PrimeScript RT reagent kit (Takara Bio, Inc.) was used to synthesize complementary DNA, which was used in the qPCR (SYBR Green PCR kit; Takara Bio, Inc.) with a 7500 Fast Real-time PCR system (Applied Biosystems; Thermo Fisher Scientific, Inc.). The RT reactions were performed as follows: 37 °C for 15 min and 85 °C for 5 s. PCR amplification was performed in a thermal cycler for 40 cycles at the following cycle conditions: 95 °C for 5 s, 60 °C for 30 s, and 72 °C for 30 s. The GAPDH expression was detected as the endogenous control. Primer sequences are shown in Additional file 1: Table S2.

### Western blotting analysis

Whole-cell lysates were prepared in RIPA lysis buffer containing 1 mM phenylmethylsulfonyl fuoride (PMSF, Cat. no. 9803, Cell Signaling Technology, Beverly, MA, USA) and protease inhibitor cocktail (Cat. no. 5872, Cell Signaling Technology, Beverly, MA, USA). The concentration of protein was quantified with the Bradford Method using Coomassie Blue Staining Solution (Beyotime Institute of Biotechnology, Haimen, China). The protein lysates were separated on 10% SDS polyacrylamide gel and transferred onto a nitrocellulose membrane. Following blocking with 5% BSA, the membrane was incubated with primary antibodies against BRD4 (Cat. no. ab128874, 1:1000; Abcam, Cambridge, MA USA), CAV-2 (Cat. no. ab2911, 1:1000; Abcam, Cambridge, MA USA), c-Myc (Cat. no. ab32072, 1:1000; Abcam, Cambridge, MA USA), PCNA (Cat. no. 13110, 1:1000; Cell Signaling Technology, Beverly, MA, USA), CDK2 (Cat. no. 2546, 1:1000; Cell Signaling Technology, Beverly, MA, USA), pRb (Cat. no. 8516, 1:1000; Cell Signaling Technology, Beverly, MA, USA), β-actin (Cat. no. sc-47778, 1:3000; Santa Cruz Biotechnology, Santa Cruz, CA, USA) and GAPDH (Cat. no. sc-47724, 1:5000; Santa Cruz Biotechnology, Santa Cruz, CA, USA) at 4 °C overnight followed by incubation with secondary antibodies.

### Colony formation assay

Briefly, ~ 800 cells were added to each well of a 6-well culture plate. After 2 weeks of incubation, cell colonies were washed twice with PBS, fixed with 4% para -formaldehyde for 15 min and then stained with 0.5% crystal violet for 30 min. The excess stained was removed. Individual clones with ≥ 50 cells were counted.

### Sulforhodamine B assay

Cells seeded in 96-well plates under different treating conditions were cultured for 24, 48, 72, and 96 h. Optical density (OD) values were measured at 560 nm by microtiter plate reader (VERSMax).

### Migration and invasion assay

The migration assay was detected by Transwell chambers (Corning, NY, USA) with or without pre-coated thin layer of Matrigel (BD Biosciences). Cells were allowed to migrate towards the bottom wells. After 24 h of incubation, the migrating cells were stained with the crystal violet solution.

### Cell apoptosis and cell cycle analysis

For cell apoptosis analysis, cells were harvested at 70–80% confluence under different treating conditions, and incubated with reagent containing Annexin V-FITC and propidium iodide (BD Biosciences) for 15 min in darkness at room temperature. Apoptotic cells were analyzed using FACSCalibur flow cytometer (BD Biosciences). For cell cycle analysis, cells were fixed in 70% ethanol at 4 °C overnight and then treated with RNase A (50 µg/ml) and stained with propidium iodide (25 µg/ml) for 30 min at 37 °C. Distribution of cell-cycle phases was determined using ModFit software (BD Biosciences).

### Chromatin immunoprecipitation (ChIP)

ChIP was performed following the manufacturer’s protocol for Simple Chip plus Enzymatic Chromatin IP Kit (Cat. no. 9005, Cell Signaling Technology, Beverly, MA, USA). The aforementioned anti-BRD4 antibody (Cat. no. ab128874, 1:50; Abcam, Cambridge, MA USA) was used for the ChIP assay. The purified DNA was analyzed by RT-qPCR. ChIP primers of CAV-2 were described in Additional file 1: Table S3.

### Dual luciferase reporter assay

Cells were plated in 96-well plates at 4 × 10^3^ cells/well, and cotransfected with BRD4-nc/BRD4-si1 and plasmid containing CAV-2 promoter regions (2000 bp, constructed by GenePharma, Shanghai) using Lipofectamine™ 2000. After 48 h, Dual Luciferase^®^ reporter assay system (Promega Corporation) was used to detect fluorescence intensity. Renilla luciferase gene was used as the internal reference to verify the transfection efficiency and calculate the relative luciferase activity as follows: Relative luciferase activity = firefly luciferase activity/Renilla luciferase activity.

### Subcutaneous xenografts models

The animal experiments were conducted according to the guidelines for animal experimentation and approved by the Experimental Animal Ethics Committee of Shanghai Jiaotong University. Stable transfected cell lines PANC-1-BRD4-nc, PANC-1-BRD4-sh1, or PANC-1-BRD4-sh2 (2 × 10^6^ cells/each) were subcutaneously implanted into BALB/c nude mice, respectively. A total of 15 nude mice were used with 5 mice in each group. Tumor size was measured every 3 days and recorded by using the following equation: Volume (mm^3^) = (length × width^2^)/2. After 30 days, the mice were sacrificed, and the tumors were excised, weighed, harvested and embedded in paraffin. The BRD4 (Cat. no. ab128874, 1:100; Abcam, Cambridge, MA USA), ki-67 (Cat. no. 9449, 1:200; Cell Signaling Technology, Beverly, MA, USA) and cleaved Caspase 3 (Cat. no. 9661, 1:100; Cell Signaling Technology, Beverly, MA, USA) expressions were detected by immunohistochemical staining respectively.

### Statistical analysis

Data were presented as the mean ± one standard deviation (SD) from at least three separate experiments. The Student’s test, one-way analysis of variance, χ^2^ test, was used for comparisons between groups as appropriate. The Kaplan–Meier method was used to estimate overall survival (OS). Univariate and multivariate COX regression analysis was performed. Those parameters with a *p*-value < 0.05 in the univariate analyses were included in a COX multivariate proportional hazards regression model. All statistical analyses were carried out using SPSS 17.0. Differences were deemed statistically significant at p < 0.05.

## Results

### BRD4 is overexpressed in PC

**Fig. 1:**
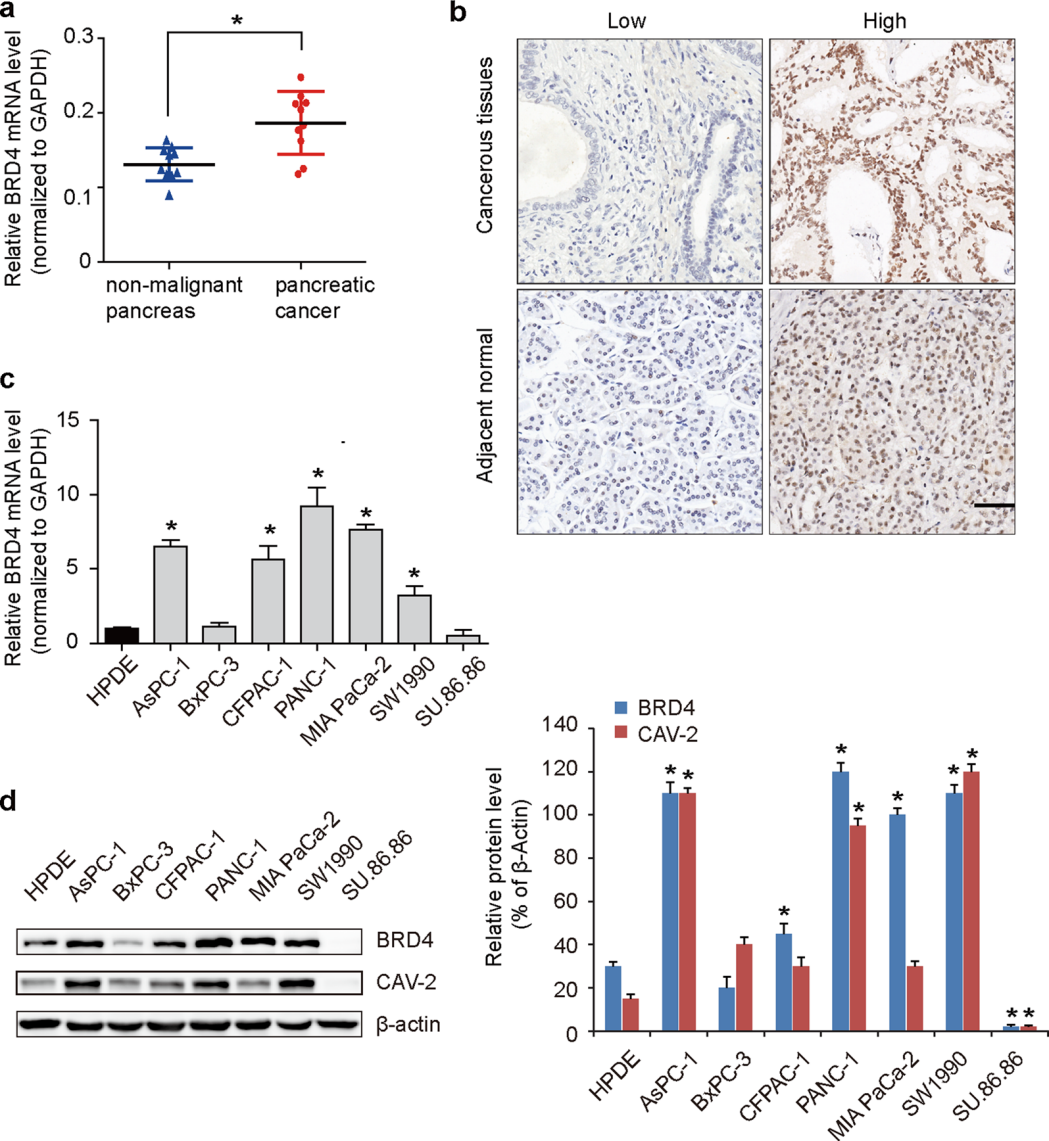
BRD4 is overexpressed in PC. **a** Compared to adjacent normal tissues, higher BRD4 mRNA expression in ten fresh samples of PC. The GAPDH expression was detected as the endogenous control. **b** Representative Immunohistochemical images of BRD4 expression, which was primarily localized in the cell nucleus. Higher BRD4 protein expression was found in PC tissues. **c** 5 of 7 PC cells lines, including AsPC-1, MIAPaCa-2, PANC-1, SW1990 and CFPAC-1 had higher BRD4 mRNA than levels observed in normal pancreatic ductal epithelial cells HPDE. The GAPDH expression was detected as the endogenous control for mRNA expression. **d** Higher BRD4 and CAV-2 protein expression in PC cell lines. BRD4 was positive correlated with CAV-2 expression. The asterisk indicates that p is less than 0.05

Ten fresh samples validated that high BRD4 mRNA expression in PC compared to adjacent normal tissues (Fig. 1a). The expression of BRD4 protein in PC tissue, evaluated using immunohistochemical staining, was primarily localized in the cell nucleus, as shown in Fig. 1b. Of the 76 paraffin-embedded tumor-tissue samples, high BRD4 protein level was found in 65.8% (50 of 76) of PC tissues, compared with only 43.4% (33 of 76) of normal tissues (p = 0.014, Table 1). Furthermore, 5 of 7 PC cells lines, including AsPC-1, MIAPaCa-2, PANC-1, SW1990 and CFPAC-1 had higher BRD4 mRNA (Fig. 1c) and protein expression (Fig. 1d) than levels observed in normal pancreatic ductal epithelial cells HPDE. These results suggest that BRD4 expression is frequently upregulated in PC.

**Table 1:** Comparisons with BRD4 and CAV-2 expression between PC and paired adjacent normal tissues

	N	BRD4 level		p-value	CAV-2 level		p-value
		Low	High		Low	High	
PC tissue	76	26	50	0.014*	34	42	< 0.001*
adjacent normal	76	43	33		65	11	

Asterisk represents the significant difference

### BRD4 promotes PC cell proliferation, migration and invasion in vitro

**Fig. 2:**
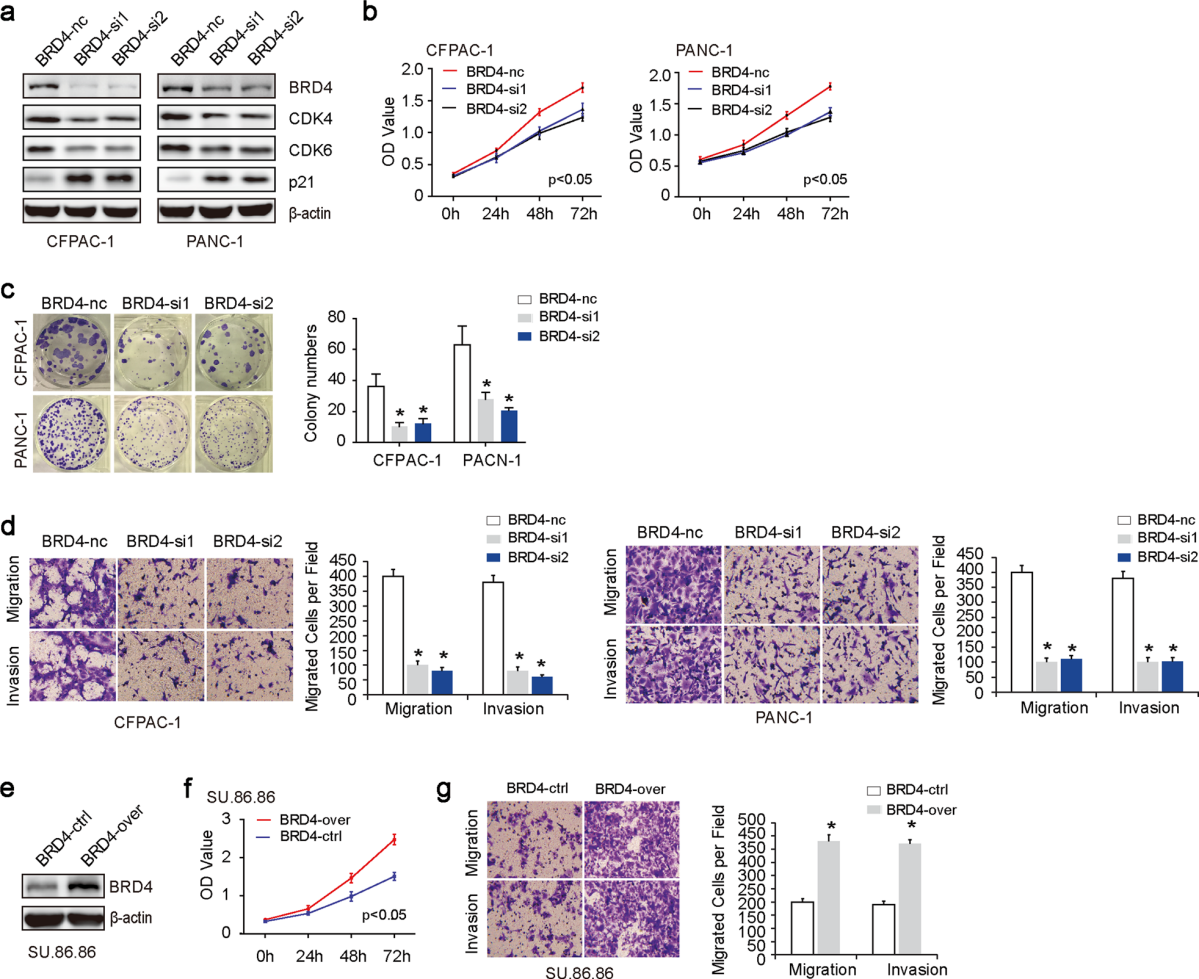
BRD4 promotes PC cell proliferation, migration and invasion in vitro. **a** Western blot was used to confirm the efficacy of BRD4 knockdown in CFPAC-1 and PANC-1 cell lines. The transfection efficiency was determined at 24 h after transfection. The results showed that G1 cell cycle CDK4 and CDK6 were decreased, while p21 was increased in response to BRD4 knockdown. **b** Sulforhodamine B assays showed that both BRD4-si1 and BRD4-si2 group significantly suppressed cell proliferation compared to BRD4-nc group. **c** Plate clone formations showed that, in comparisons with BRD4-nc group, both BRD4-si1 and BRD4-si2 group significantly suppressed clone formations. **d** Migration assay showed that both BRD4-si1 and BRD4-si2 group significantly suppressed cell migration and invasion compared to BRD4-nc group. **e** Western blot was used to confirm the efficacy of BRD4 overexpression in su.86.86. **f** Sulforhodamine B assays showed that BRD4-over group significantly promoted cell proliferation compared to BRD4-ctrl group. **g** Migration assay showed that BRD4-over group significantly increased cell migration and invasion compared to BRD4-ctrl group. The asterisk indicates that p is less than 0.05

Given the fact that CFPAC-1 and PANC-1 have relatively higher endogenous BRD4 expression, we chose them for BRD4 silencing experiments. Western blot was used to confirm the efficacy of BRD4 knockdown (Fig. 2a). Sulforhodamine B assays (Fig. 2b) and plate clone formations (Fig. 2c) revealed that downregulation of BRD4 expression significantly suppressed cell proliferation. Western blot results showed that G1 cell cycle CDK4 and CDK6 were decreased, while p21 was increased in response to BRD4 knockdown (Fig. 2a). Migration assay revealed that inhibition of BRD4 significantly decreased the migration and invasive capacities in CFPAC-1 and PANC-1(Fig. 2d). Correspondingly, su.86.86 was used for BRD4 overexpression experiment (Fig. 2e). The results showed that BRD4 overexpression promoted cell proliferation (Fig. 2f), migration and invasion (Fig. 2g) in vitro.

### BRD4 increases PC cell growth in vivo

**Fig. 3:**
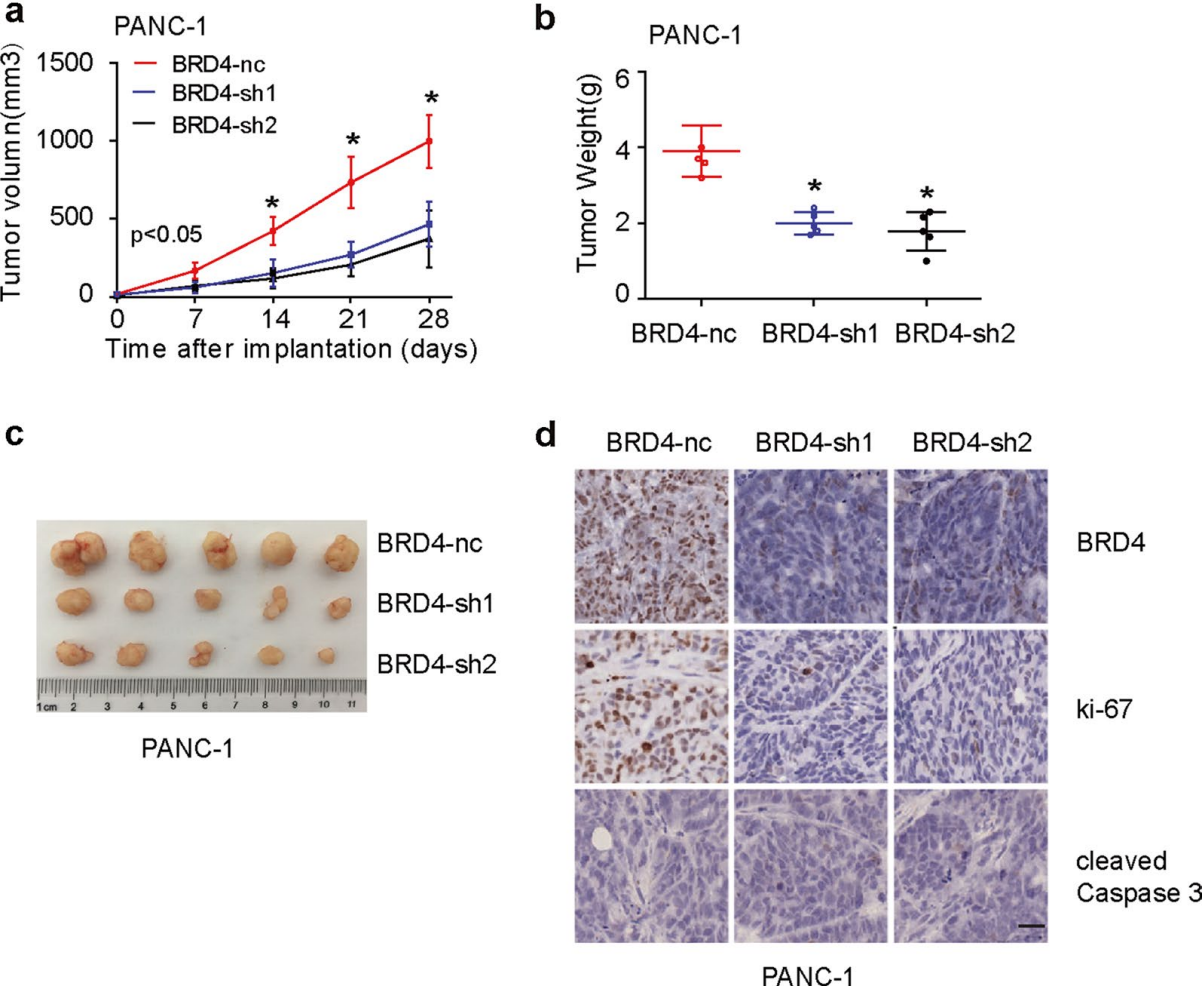
BRD4 increases PC cell growth in vivo. **a**–**c** Subcutaneous tumor transplantation test showed that, compared with PANC-1/BRD4-nc, PANC-1/BRD4-sh1 and PANC-1/BRD4-sh2 groups had slower growth rate (**a**) and tumor weight (**b**, **c**). **d** Representative immunohistochemical images for BRD4, ki67 and cleaved Caspase3 expression. In comparisons with PANC-1/BRD4-nc, both PANC-1/BRD4-sh1 and PANC-1/BRD4-sh2 groups had lower BRD4 and ki67 expression. However, cleaved Caspase 3 was negatively expressed in all groups. Scale bar, 50 μm. The asterisk indicates that p is less than 0.05

To further investigate whether interference with BRD4 could inhibit tumor growth in vivo, we constructed stable inhibition of BRD4 cell line in PANC-1. Subcutaneous tumor transplantation test showed that, compared with PANC-1/BRD4-nc, PANC-1/BRD4-sh1 and PANC-1/BRD4-sh2 groups had slower growth rate (Fig. 3a) and tumor weight (Fig. 3b, c). Immunohistochemical results suggested that BRD4 and ki67 expressions were lower in BRD4 knockdown groups (Fig. 3d). However, cleaved Caspase 3 was negatively expressed in all groups (Fig. 3d), indicating that the mechanism of BRD4 promoting cell growth may not be related to regulation of cell apoptosis.

### CAV-2 is a downstream target of BRD4

**Fig. 4:**
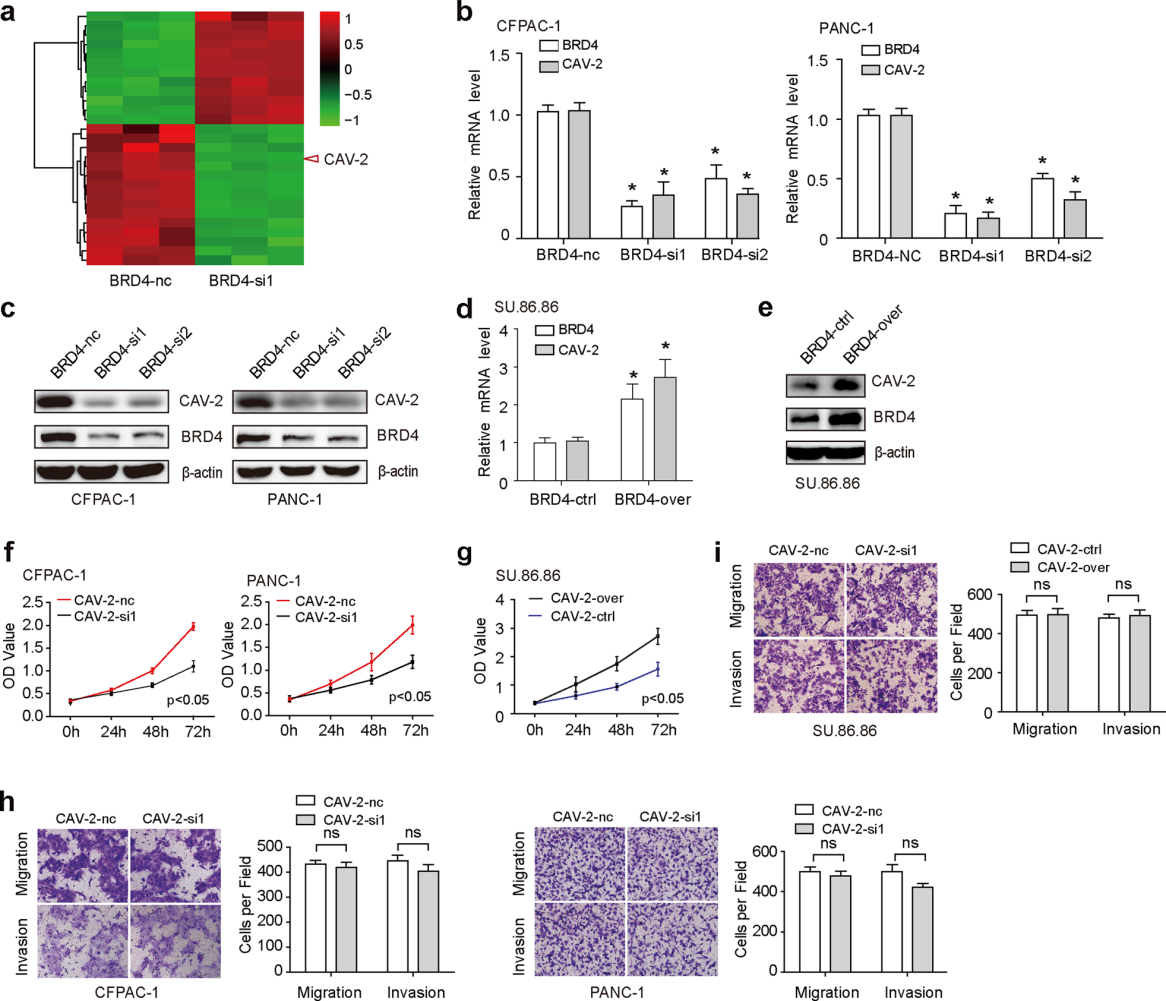
CAV-2 is a downstream target of BRD4. **a** RNA sequence was employed to identify the downstream target of BRD4. The results showed that there were 27 differentially expressed gene, 12 upregulations and 15 downregulation. Among them, the downregulation of CAV-2 was the second most. **b**, **c** The CAV-2 mRNA (**b**) and protein (**c**) level in both BRD4-si1 and BRD4-si2 group was down-regulated. **d**, **e** Compared to BRD4-ctrl group, the CAV-2 mRNA (**d**) and protein (**e**) in BRD4-over group was up-regulated. **f** Sulforhodamine B assays showed that both CAV-2-si1 and CAV-2-si2 group significantly suppressed cell proliferation compared to CAV-2-nc group. **g** Sulforhodamine B assays showed that CAV-2-over group significantly promoted cell proliferation compared to BRD4-ctrl group. **h** Migration assay showed that both CAV-2-si1 and CAV-2-si2 group did not significantly suppressed cell migration and invasion compared to CAV-2-nc group. **i** Migration assay showed that BRD4-over group did not significantly increased cell migration and invasion compared to BRD4-ctrl group. The asterisk indicates that p is less than 0.05

RNA sequence was employed to identify the downstream target of BRD4. We found that there were 27 differentially expressed gene, 12 up regulations and 15 down regulations (Fig. 4a, Additional file 1: Table S4). Among them, the downregulation of Caveolin-2 (CAV-2) was shown to be the second most. CAV-2 is a member of the caveolae family which has an essential role in intracellular cell transport and signal transduction. Higher level of CAV-2 has been associated with different types of cancer progression [16–18]. In our study, after silence of BRD4 expression in CFPAC-1 and PANC-1, the CAV-2 mRNA (Fig. 4b) and protein (Fig. 4c) level was down-regulated. While after overexpression of BRD4 in su.86.86, the CAV-2 mRNA (Fig. 4d) and protein (Fig. 4e) was up-regulated. Further biological research showed that CAV-2 promoted cell proliferation in PC (Fig. 4f, g), but not cell migration and invasion (Fig. 4h, i).

### CAV-2 overexpression partially restores BRD4-siRNA mediated cell proliferation

**Fig. 5:**
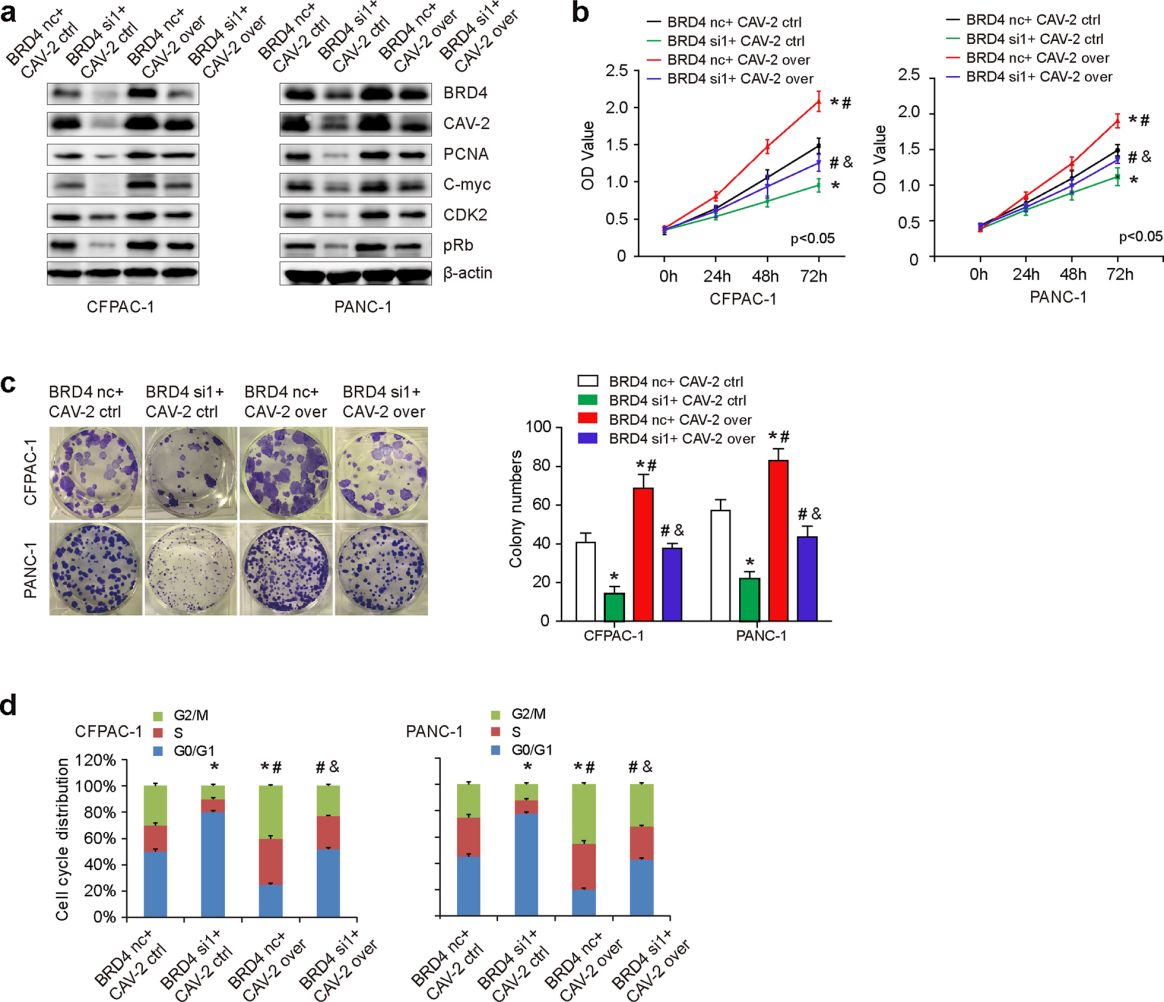
CAV-2 overexpression partially restores BRD4-siRNA mediated cell proliferation. **a** Western blot was employed to detect BRD4, CAV-2, PCNA, C-myc, CDK2, and pRb expression when cotransfected CFPAC-1 and PANC-1 cells with BRD4 nc/siRNA and CAV-2 ctrl/over plasmid. BRD4 decreased PCNA, C-myc, CDK2, and pRb expression. CAV-2 overexpression can partially reverse the protein expression of BRD4 suppression. **b**, **c.** Sulforhodamine B assays (**b**) and plate clone formations (**c**) showed that CAV-2 overexpression partially reversed decreased cell proliferation of BRD4 knockdown. **d** Cell cycle experiments showed that suppression of BRD4 induced G1 phase arrest. CAV-2 overexpression can partially reverse the effects of BRD4 knockdown. The asterisk indicates that p is less than 0.05

To further examine if the effects of BRD4 on cell proliferation were partially mediated by CAV-2, we cotransfected CFPAC-1 and PANC-1 cells with BRD4 nc/siRNA and CAV-2 ctrl/over plasmid (Fig. 5a). Sulforhodamine B assays (Fig. 5b) and plate clone formations (Fig. 5c) showed that CAV-2 overexpression partially reversed decreased cell proliferation of BRD4 knockdown. Further cell cycle (Fig. 5d) experiments showed that suppression of BRD4 induced G1 phase arrest. CAV-2 overexpression can partially reverse the effects of BRD4 knockdown. Meanwhile, we also detected genes related to cell proliferation and cycle, and found that reduced BRD4 decreased PCNA, C-myc, CDK2, and pRb expression. CAV-2 overexpression can partially reverse the protein expression of BRD4 suppression (Fig. 5a).

### BRD4 regulates CAV-2 transcriptional activity

**Fig. 6:**
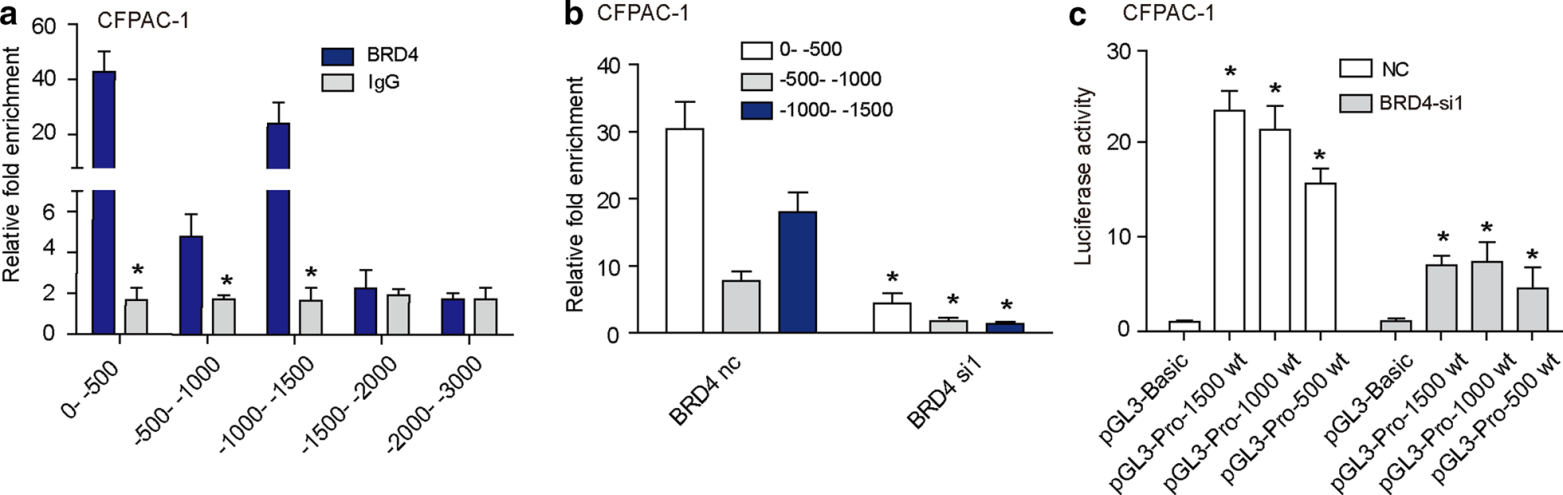
BRD4 regulates CAV-2 transcriptional activity. **a** ChIP results showed that BRD4 can directly bind to the promoter region (0 ~ − 500 bp, − 500 ~ − 1000 bp and − 1000 ~ − 1500 bp) of CAV-2. **b** Compared to BRD4-nc group, BRD4-si1 exhibited a reduced BRD4 recruitment to CAV-2 promoter. **c** Luciferase reporter experiment suggested that BRD4 had transcriptional activity at the − 1500 ~ 0 bp promoter region of CAV-2. After interfering with BRD4 expression, the transcriptional activation of CAV-2 could be inhibited. The asterisk indicates that p is less than 0.05

Previous data showed that BRD4 regulated the expression of CAV-2 at the mRNA level, suggesting that this down-regulation may occur at the transcriptional level. Therefore, we examined the binding of BRD4 to the promoter region of the CAV-2 gene in CFPAC-1. Five pairs segmented primers for the CAV-2 promoter region were designed (0 ~ − 500 bp, − 500 ~ − 1000 bp, − 1000 ~ − 1500 bp, − 1500 ~ − 2000 and − 2000 ~ − 3000 bp). ChIP results showed that BRD4 can directly bind to the promoter region (0 ~ − 500 bp, − 500 ~ − 1000 bp and − 1000 ~ − 1500 bp) of CAV-2 (Fig. 6a). Further, Compared to BRD4-nc group, BRD4-si1 exhibited a reduced BRD4 recruitment to CAV-2 promoter (Fig. 6b). Luciferase reporter experiment suggested that BRD4 had transcriptional activity at the − 1500 ~ 0 bp region of CAV-2 (Fig. 6c). After interfering with BRD4 expression, the transcriptional activation of CAV-2 could be inhibited (Fig. 6c).

### BRD4 and CAV-2 protein levels correlate with pathologic features and OS of PC

The correlation between BRD4 and CAV-2 protein levels and clinicopathological characteristics of PC were further evaluated. As shown in Table 2, a high level of BRD4 was positively correlated with clinical stage (p = 0.024), lymph nodes metastasis (p = 0.022), nervous invasion (p = 0.022), and vessel invasion (p = 0.025). However, it was not associated with the age (p = 0.131), gender (p = 0.532), invasion depth (p = 0.205), distant metastasis (p = 1.000), tumor differentiation (p = 0.424) and tumor location (p = 0.106). A high level of CAV-2 was positively correlated with clinical stage (p < 0.001), invasion depth (p = 0.015), lymph nodes metastasis (p = 0.004), and tumor differentiation (p = 0.040).

**Table 2:** Correlation between the clinicopathological characteristics and BRD4 and CAV-2 expression in PC

Characteristics	N	BRD4 level		p-value	CAV-2 level		p-value
		Low (26)	High (50)		Low (34)	High (42)	
Age (years)							
≤ 60	23	5	18	0.131	8	15	0.250
> 60	53	21	32		26	27	
Gender							
Male	49	18	31	0.532	22	27	0.970
Female	27	8	19		12	15	
Clinical stage							
Early stages (≤ I)	12	8	4	0.024*	11	1	< 0.001*
Advanced stages (> I)	64	18	46		23	41	
Invasion depth							
T1 + T2	17	8	9	0.205	12	5	0.015*
T3 + T4	59	18	41		22	37	
Lymph nodes metastasis							
N0 (negative)	33	16	17	0.022*	21	12	0.004*
N1 (positive)	43	10	33		13	30	
Distant metastasis							
M0 (absent)	72	25	47	1.000	33	39	0.765
M1 (present)	4	1	3		1	3	
Tumor differentiation							
Well, moderate	51	19	32	0.424	27	24	0.040*
Poor	25	7	18		7	18	
Tumor location							
Head, neck	39	10	29	0.106	14	25	0.112
Body, tail	37	16	21		20	17	
Nervous invasion							
Negative	25	13	12	0.022*	14	11	0.167
Positive	51	13	38		20	31	
Vessel invasion							
Negative	65	26	39	0.025*	32	33	0.112
Positive	11	0	11		2	9	

Asterisk represents the significant difference

**Fig. 7:**
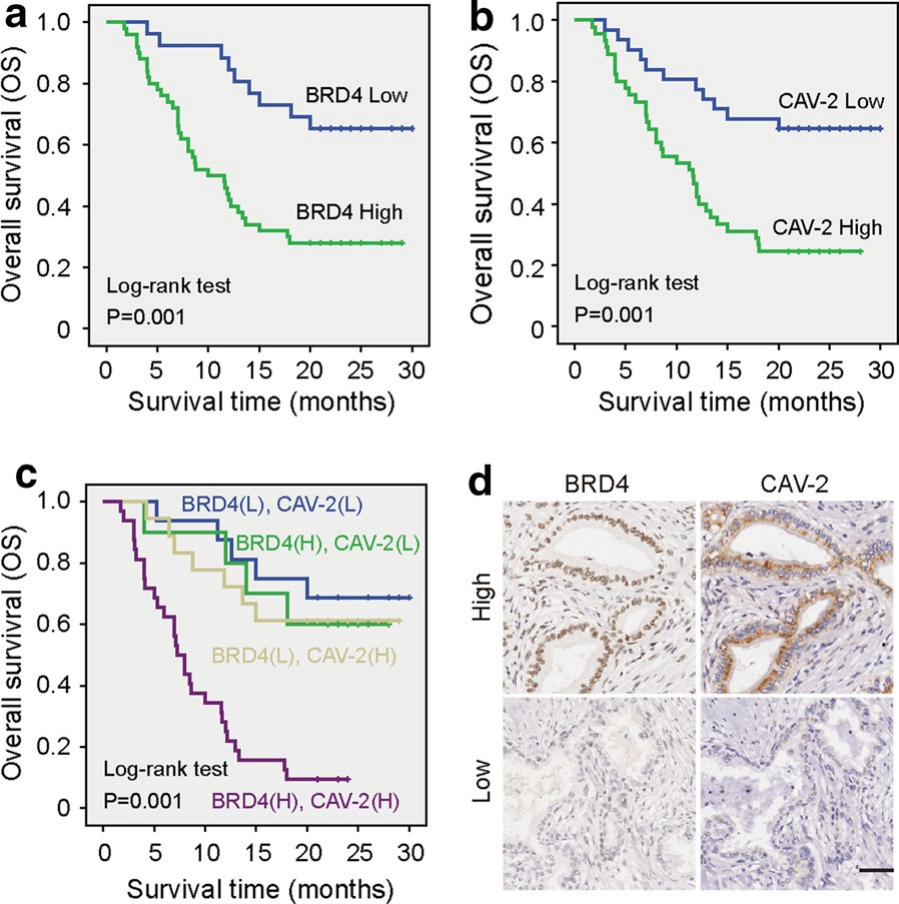
Association between BRD4 and CAV-2 expression and prognosis in PC patients. **a** The log-rank test showed that patients with PC tumors expressing high BRD4 levels had significantly lower OS than those expressing low BRD4. **b** The patients expressing high CAV-2 levels had significantly lower OS than those expressing low CAV-2. **c** The log-rank test showed that BRD4 (high)/CAV-2 (high) correlated with shorter OS of PC patients compare to that of other groups, including BRD4 (low)/CAV-2 (low), BRD4 (low)/CAV-2 (high) and BRD4 (high)/CAV-2 (low). **d** Serial histological sections suggested a positive correlation between BRD4 and CAV-2 expression

Kaplan–Meier analysis revealed that patients with higher BRD4 (Fig. 7a) and CAV-2 expression (Fig. 7b) possessed worse prognosis. Univariate survival analysis revealed that, in addition to clinical stage, tumor differentiation, nervous invasion and vessel invasion, high BRD4 (HR = 3.321, p = 0.001) and CAV-2 expression (HR = 3.666, p < 0.001) predicted poor prognosis (Table 3). Multivariate analysis showed that high BRD4 (HR = 2.244, p = 0.047) and CAV-2 expression (HR = 2.370, p = 0.026) were correlated with OS (Table 3). To further investigate the association of survival time with BRD4 and CAV-2 expression, a final concomitant model was constructed. As shown in Fig. 7c, the log-rank test showed that high co-expression of BRD4 and CAV-2 proteins correlated with shorter OS of PC patients (p = 0.001). Moreover, Spearman correlation analysis revealed a positive correlation between BRD4 and CAV-2 expression (r = 0.244, p = 0.034; Fig. 7d, Table 4).

**Table 3:** Summary of univariate and multivariate Cox regression analysis of overall survival

Characteristics	Univariate analysis			Multivariate analysis		
	HR	95% CI	p-value	HR	95% CI	p-value
BRD4						
Low	1			1		
High	3.321	1.592–6.930	0.001*	2.244	1.009–4.990	0.047*
CAV-2						
Low	1			1		
High	3.666	1.881–7.143	< 0.001*	2.370	1.108–5.071	0.026*
Age (years)						
≤ 60	1					
> 60	0.765	0.415–1.409	0.390			
Gender						
Male	1					
Female	1.116	0.600–2.076	0.728			
Clinical stage						
Early stages (≤ I)	1			1		
Advanced stages (> I)	4.059	1.255–13.123	0.019*	2.120	0.560–8.025	0.269
Invasion depth						
T1 + T2	1					
T3 + T4	1.937	0.864–4.342	0.109			
Lymph nodes metastasis						
N0 (negative)	1					
N1 (positive)	1.264	0.699–2.284	0.439			
Distant metastasis						
M0 (Absent)	1					
M1 (Present)	0.597	0.144–2.468	0.476			
Tumor differentiation						
Well, moderate	1			1		
Poor	1.963	1.084–3.553	0.026*	1.798	0.916–3.530	0.088
Tumor location						
Head, neck	1					
Body, tail	1.541	0.854–2.779	0.151			
Nervous invasion						
Negative	1			1		
Positive	2.018	1.020–3.993	0.044*	0.995	0.470–2.106	0.990
Vessel invasion						
Negative	1			1		
Positive	4.107	1.978–8.528	< 0.001*	4.100	1.715–9.804	0.002*

Asterisk represents the significant difference

**Table 4:** Correlation between BRD4 and CAV-2 expression in PC

	N	BRD4 level		Correlation coefficient	p-value
		Low	High		
CAV-2 (low)	34	16	18	0.244	0.034*
CAV-2 (high)	42	10	32		

Asterisk represents the significant difference

## Discussion

The bromodomain and extra-terminal domain (BET) family of proteins, especially BRD4 has emerged as regulating multiple genes expression involved in cell cycle, cell growth and inflammation due to their important roles in epigenetic regulation [19, 20]. Unbiased genetic screening for a variety of cancers has shown that BRD4 is critical for cell survival and a promising anticancer target [21], such as acute myeloid leukemia [22], glioblastoma [23] and lung adenocarcinoma [24]. In contrast, BRD4 is downregulated in colon [25] and breast cancers [26]. Therefore, BRD4 may serve as a tumor suppressor in these cancers. These conflicting results suggest that the function of BRD4 in different tissues depends on the type of cancer. Our data showed that BRD4 was overexpressed in PC. Biological results demonstrated that BRD4 functioned as tumor promoter, facilitated PC cell proliferation, migration and invasion in vitro and in vivo, consistent with previous research [27, 28].

However, how BRD4 regulates cell growth, migration and invasion is not clear. Sahai et al. [27] found that BET inhibitors and BRD4 siRNA inhibited HMGA2, a structural protein that regulates chromatin status and also contributed to chemoresistance. Hogg et al. [29] reported that suppression of BRD4 inhibited the transcription of PD-L1 independent of Myc expression. The therapeutic effect of BET inhibitor combined with immune modulating therapy was better. Yang et al. [30] revealed that VDAC1 was regulated by BRD4 and contributed to JQ1 resistance in breast cancer. Qin et al. [31] demonstrated that BRD4 promoted progression and metastasis of gastric cancer by acetylation-dependent stabilization of Snail. Further genome-wide transcriptome analysis identified that BRD4 and Snail modulated partially shared metastatic gene signature. Otto et al. [32] found that inhibition and degradation of BRD4 are powerful tools for reducing MYC expression and CRC cell proliferation. Jin et al. [33] revealed that deubiquitinating BRD4 promoted BET inhibitor resistance and cancer Progression. Therefore, in-depth understanding of the molecular mechanism of BRD4 in PC can help to find the genes that may be regulated by BRD4, and the design of targeted inhibitors for both may be a new therapeutic direction.

We further identified the downstream targets of BRD4 by RNA sequencing. There were 27 differentially expressed genes, 12 up-regulated and 15 down-regulated. Among them, the downregulation of CAV-2 was shown to be the second most. CAV-2 is a member of the caveolae family and plays a vital role in intracellular cell transport and signal transduction [34]. Higher level of CAV-2 has been associated with progression of different types of cancer such as lung [16] and breast cancer [35]. In pancreatic cancer, CAV-2 overexpression might be involved in the metastatic potential and associated with poorer prognosis with shorter overall survival and disease-free survival [36]. Our data showed that knockdown of CAV-2 inhibited cell proliferation, but not cell migration and invasion in PC. Further, CAV-2 overexpression can partially reverse the inhibitory effects of BRD4 knockdown. ChIP assay and dual luciferase reporter assay revealed BRD4 could bind to the promoter region of CAV-2 and upregulate CAV-2 expression. Interestingly, we found that the level of BRD4 protein increased after CAV-2 overexpression. Xu et al. [37] found that targeting BRD4 through the JAK2/STAT3 pathway could inhibit tumor cell proliferation, migration, and invasion. Kwon et al. [38] revealed that phosphotyrosine-caveolin-2 was a novel regulator for transcriptional activation of STAT3. Therefore, we speculate that CAV-2 overexpression results in activation of the STAT3 signaling, leading to BRD4 upregulation. This hypothesis needs our further research. In addition, gene ontology analyses results of BRD4 knockdown were shown in Additional file 2: Fig. S1 and Additional file 3: Fig. S2. The next study will focus on the molecular mechanism by which BRD4 regulates metastasis in pancreatic cancer.

The correlation between BRD4 and CAV-2 protein levels and clinicopathological characteristics of PC were further evaluated. BRD4 was positively correlated with clinical stage, lymph nodes metastasis, nervous invasion, and vessel invasion. And CAV-2 was positively correlated with clinical stage, invasion depth, lymph nodes metastasis, and tumor differentiation. Survival analysis results showed that patients with higher BRD4 and CAV-2 expression possessed worse prognosis. Multivariate COX analysis revealed that BRD4 and CAV-2 were independent factors in determining prognosis of patients with PC. Moreover, there was a positive correlation between BRD4 and CAV-2 expression.

## Conclusion

In sum, our findings reveal the oncogenic effects of BRD4 in PC and elucidate a possible mechanism by which BRD4 and CAV-2 act to enhance PC growth. Given that overexpression of both BRD4 and CAV-2 are observed in PC, targeting the BRD4-CAV-2 interaction by developed BET inhibitors may be applicable to prevent tumor growth in PC.

## Supplementary information

**Additional file 1: Table S1.** Detailed clinical information and follow-up data of 76 patients with PC. **Table S2.** The primer sequence of BRD4, CAV-2 and GAPDH. **Table S3.** The ChIP primer sequence of CAV-2. **Table S4.** Differentially expressed genes following BRD4 knockdown.

**Additional file 2: Fig. S1.** The diagram of gene oncology enrichment.

**Additional file 3: Fig. S2.** The diagram of gene oncology classification.

## Data Availability

All data generated during this study are included in this article.

## Abbreviations

BET: Bromodomain and extra-terminal domain
PC: Pancreatic cancer
TMA: Tissue microarray
RT-qPCR: Real-time reverse transcription polymerase chain reaction
ChIP: Chromatin immunoprecipitation
SD: Standard deviation
OS: Overall survival
