# Supplementary material for: Caveolin-2 is regulated by BRD4 and contributes to cell growth in pancreatic cancer
Source: Cancer Cell Int. 2020 Feb 18;20:55. doi: 10.1186/s12935-020-1135-0 (PMC7029443; doi:10.1186/s12935-020-1135-0)
Supplement: Supplementary file 1 — Additional file 1: Table S1. Detailed clinical information and follow-up data of 76 patients with PC. Table S2. The primer sequence of BRD4, CAV-2 and GAPDH. Table S3. The ChIP primer sequence of CAV-2. Table S4. Differentially expressed genes following BRD4 knockdown. [file 12935_2020_1135_MOESM1_ESM.docx]

**Table S1** Detailed clinical information and follow-up data of 76 patients with PC

| **Characteristics** | **Categories** | **Number** |
| --- | --- | --- |
| OS median (range, months) | | 15.6 (2-30) |
| Age median (range, years) |  | 61.6 (31-78) |
| Gender |  |  |
|  | Male | 49 |
|  | Female | 27 |
| Clinical stage |  |  |
|  | Early stages (≤ I) | 12 |
|  | Advanced stages (>I) | 64 |
| Invasion depth |  |  |
|  | T1 | 2 |
|  | T2 | 15 |
|  | T3 | 59 |
|  | T4 | 0 |
| Lymph nodes metastasis |  |  |
|  | N0(negative) | 33 |
|  | N1(positive) | 43 |
| Distant metastasis |  |  |
|  | M0(Absent) | 72 |
|  | M1(Present) | 4 |
| Tumor differentiation |  |  |
|  | Well | 8 |
|  | moderate | 43 |
|  | Poor | 25 |
| Tumor location |  |  |
|  | Head, neck | 39 |
|  | Body, tail | 37 |
| Nervous invasion |  |  |
|  | Negative | 25 |
|  | Positive | 51 |
| Vessel invasion |  |  |
|  | Negative | 65 |
|  | Positive | 11 |

**Table S2** The primer sequence of BRD4, CAV-2 and GAPDH

| Gene | Primer sequence (5’-3’) |
| --- | --- |
| BRD4 | Forward: 5’-TGGAAGAAGGGGAACAAATG- 3’ |
|  | Reverse: 5’- CAAGGCTGCGAGATACACAA -3’ |
| CAV-2 | Forward: 5’-AGTTCCTGACGGTGTTCCTG-3’ |
|  | Reverse: 5’-GCAGAACCATTAGGCAGGTC-3’ |
| GAPDH | Forward: 5’- TGAACGGGAAGCTCACTGG -3’ |
|  | Reverse: 5’- TCCACCACCCTGTTGCTGTA -3’ |

**Table S3** The ChIP primer sequence of CAV-2

| CAV-2 promoter region | Primer sequence |
| --- | --- |
| -3000~-2000 | Forward: 5’-AGAGGCTCAATACCAGCACCAT-3’ |
|  | Reverse: 5’- TGGAGGGAGGGAACCTTGTG-3’ |
| -2000 ~ -1500 | Forward: 5’-TGGGTAAGCAGGGTGATGTCT-3’ |
|  | Reverse: 5’-GGTGGGAGGCAAAGTTCAAT-3’ |
| -1500 ~ -1000 | Forward: 5’-TGCACCTCAAGCCTAGTTCTCAT-3’ |
|  | Reverse: 5’- GAATTAGCCTGCTGACCACTCAC-3’ |
| -1000 ~ -500 | Forward: 5’- AGGCTGAGGCAGGAGAATCG -3’ |
|  | Reverse: 5’- GGCAGAGTTTCGCTCTTGTTG-3’ |
| -500 ~ 0 | Forward: 5’- AGAGGCTCAATACCAGCACCAT-3’ |
|  | Reverse: 5’- TGGAGGGAGGGAACCTTGTG-3’ |

**Table S4** Differentially expressed genes following BRD4 knockdown

| Gene | log2FC | P-value | Q-value | Up/Down |
| --- | --- | --- | --- | --- |
| ATL3 | -1.81306 | 4.76E-55 | 6.61E-51 | Down |
| CAV2 | -1.76618 | 8.74E-55 | 8.09E-51 | Down |
| ATP5G1 | -2.1967 | 1.28E-53 | 8.86E-50 | Down |
| PLOD2 | -1.65837 | 6.09E-51 | 3.38E-47 | Down |
| PLOD2 | -1.65837 | 6.09E-51 | 3.38E-47 | Down |
| API5 | -1.51935 | 1.47E-40 | 3.55E-37 | Down |
| TGFB2 | -1.41212 | 2.85E-35 | 4.95E-32 | Down |
| POMP | -1.71256 | 1.87E-33 | 2.6E-30 | Down |
| ALDH1A3 | -1.32611 | 4.59E-35 | 7.5E-32 | Down |
| HSPA4L | -1.47928 | 3.22E-26 | 2.48E-23 | Down |
| NETO2 | -1.15518 | 3.78E-24 | 2.5E-21 | Down |
| ARHGDIA | -1.29152 | 1.54E-23 | 9.7E-21 | Down |
| CDK13 | -1.0684 | 1.15E-21 | 5.39E-19 | Down |
| SHH | -1.26546 | 5.13E-14 | 9.82E-12 | Down |
| GATA4 | -1.05549 | 5.22E-14 | 9.86E-12 | Down |
| CTGF | 1.543237 | 1.91E-44 | 5.88E-41 | Up |
| SETD7 | 1.612294 | 4.12E-43 | 1.14E-39 | Up |
| TPM4 | 1.893186 | 1.53E-40 | 3.55E-37 | Up |
| PODXL | 1.43758 | 1.19E-35 | 2.21E-32 | Up |
| LASP1 | 1.356538 | 1.08E-31 | 1.2E-28 | Up |
| EMC6 | 1.660862 | 1.51E-29 | 1.4E-26 | Up |
| ANKRD1 | 1.337028 | 3.38E-29 | 3.03E-26 | Up |
| BCL2L1 | 1.149856 | 2.76E-24 | 1.92E-21 | Up |
| GANAB | 1.306094 | 6.12E-28 | 5.31E-25 | Up |
| GXYLT1 | 1.16548 | 1.91E-22 | 9.82E-20 | Up |
| HIST1H2BD | 1.970342 | 2.79E-19 | 1.05E-16 | Up |
| COL13A1 | 1.026027 | 6.56E-13 | 1.04E-10 | Up |
