# Supplementary figures and images for: Caveolin-2 is regulated by BRD4 and contributes to cell growth in pancreatic cancer
Source: Cancer Cell Int. 2020 Feb 18;20:55. doi: 10.1186/s12935-020-1135-0 (PMC7029443; doi:10.1186/s12935-020-1135-0)

# Top 30 of GO Enrichment

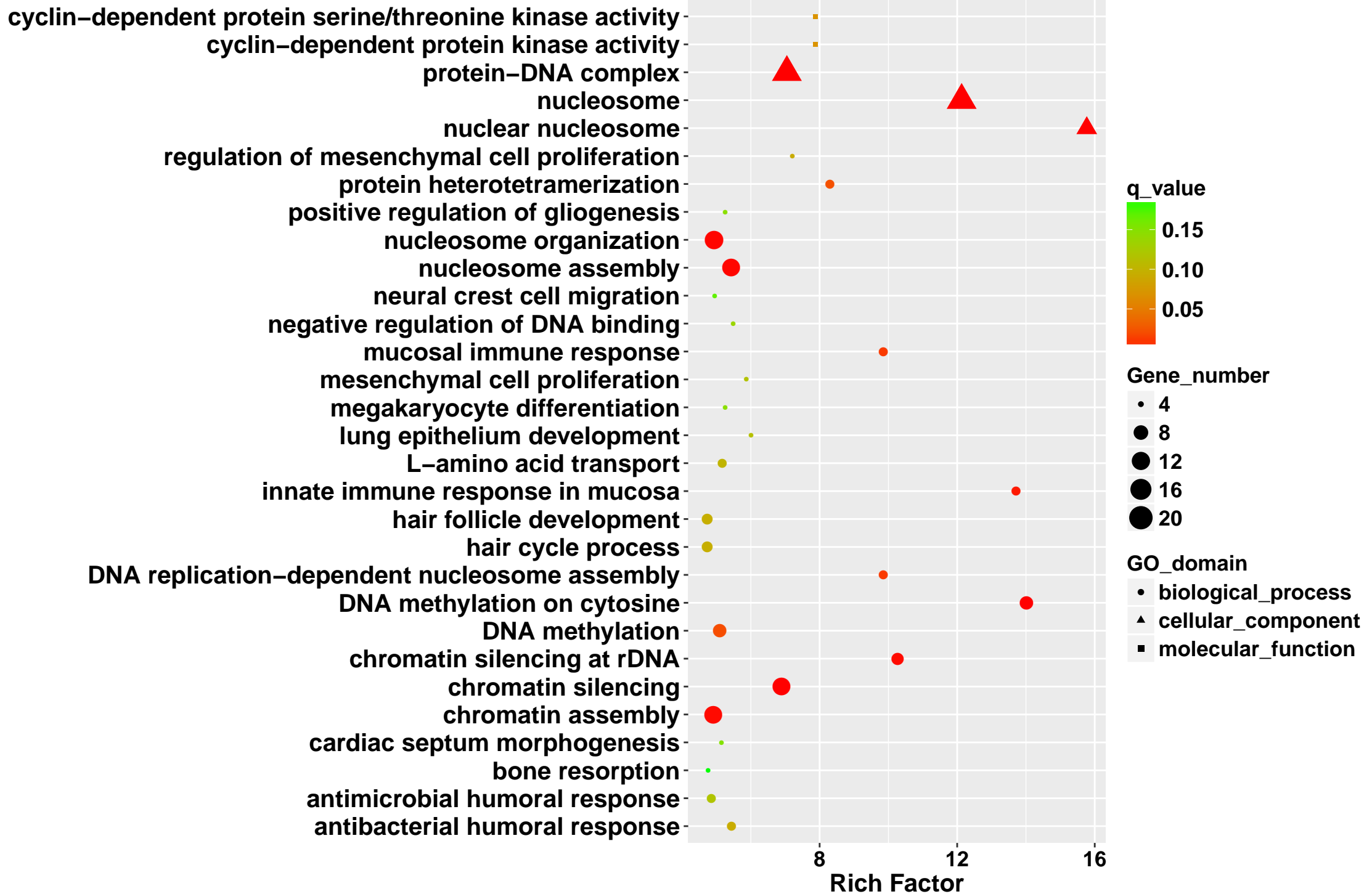

Supplement: Supplementary file 2 — Additional file 2: Fig. S1. The diagram of gene oncology enrichment. [file 12935_2020_1135_MOESM2_ESM.pdf]

# GO Classification

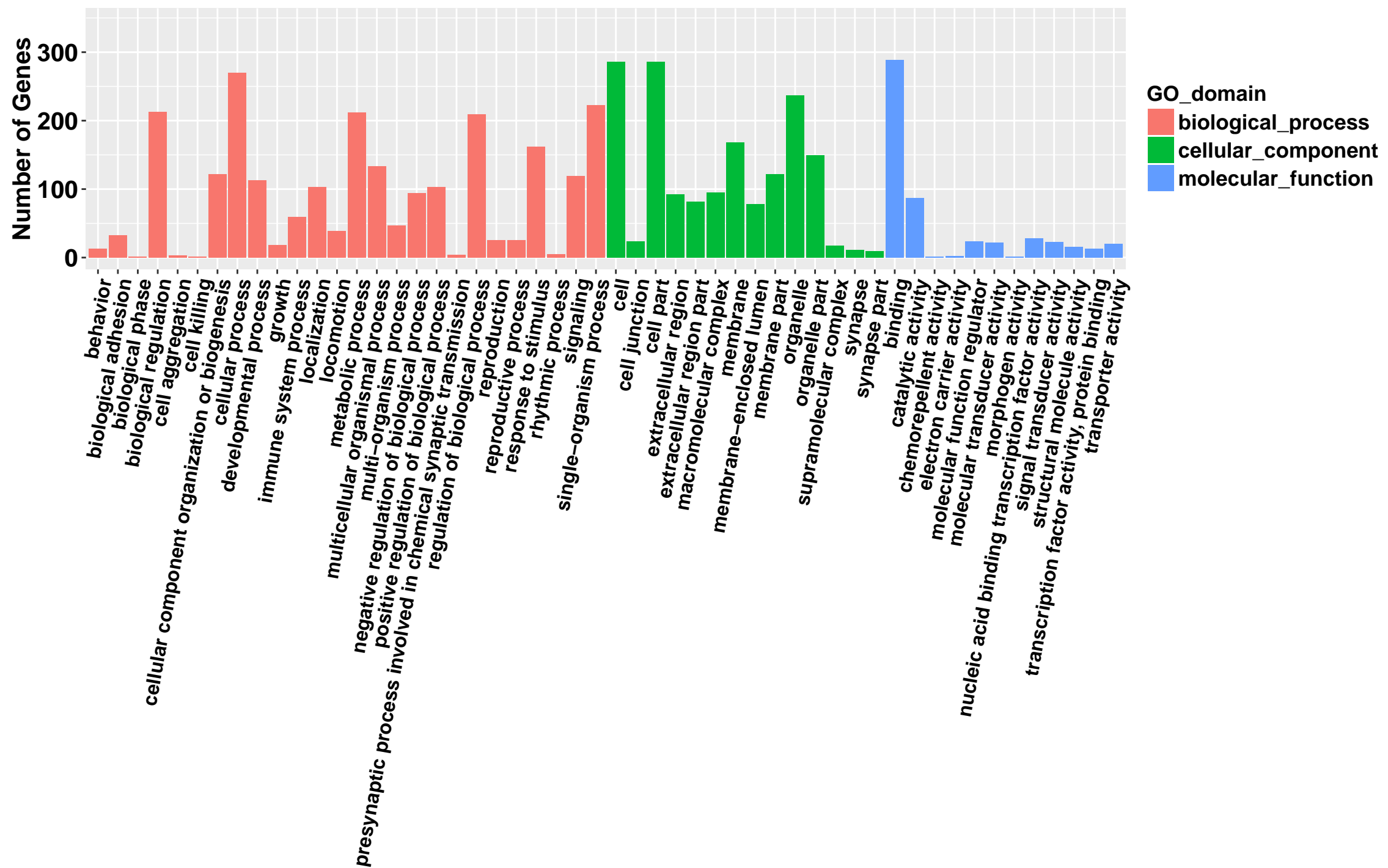

Supplement: Supplementary file 3 — Additional file 3: Fig. S2. The diagram of gene oncology classification. [file 12935_2020_1135_MOESM3_ESM.pdf]
